# Psychiatriekritik auf die Straße bringen. Mad Pride-Paraden und Blaue Karawane als Arbeit an der Multiplizität

**DOI:** 10.1007/s00048-024-00404-2

**Published:** 2024-11-14

**Authors:** Ronda Ramm, Beate Binder, Francis Seeck

**Affiliations:** 1 Institut für Europäische Ethnologie, Humboldt-Universität zu Berlin, Berlin, Deutschland; 2 Fakultät Sozialwissenschaften, Technische Hochschule Nürnberg, Nürnberg, Deutschland

**Keywords:** Psychiatriekritik, Performance, Deutschland, Soziale Bewegungen, Demonstration, Protestpraxis, Critique of psychiatry, Performance, Germany, Social movements, Demonstration, Protest practice

## Abstract

Im Mittelpunkt dieses Beitrags stehen performative Artikulationen von Psychiatriekritik bei Demonstrationen: Wir betrachten Mad Pride-Paraden, die in verschiedenen deutschen Städten seit 2013 durchgeführt werden, in Relation zu Aktionen der Bremer „Blauen Karawane“ – einer Bewegung, die in den 1980er Jahren im Zuge der Auflösung einer psychiatrischen Großklinik entstand. Obwohl sie in unterschiedlichen zeitlich-lokalen Kontexten situiert sind, setzen beide auf Formen des Straßenprotests, um die Grenzziehung zwischen „normal“ und „verrückt“ infrage zu stellen und die gleichberechtigte Anerkennung psychischer Alterität voranzubringen. Eine detaillierte Untersuchung der Aktionsformen unterstreicht die Bedeutung von karnevaleskem Feiern, Provokationen und Spektakel für beide Formen der Psychiatriekritik. Wir argumentieren, dass diese Formen Kritik erfahrbar und zugleich Entwürfe einer anderen – besseren – Gesellschaft greifbar machen. Um diese Aspekte herauszuarbeiten, greifen wir auf queer- und gendertheoretische Überlegungen sowie auf Ansätze der Performance Studies zurück. Die Betrachtung von Mad Pride-Paraden und Blauer Karawane in ihren jeweiligen Kontexten offenbart Ähnlichkeiten in der Art und Weise, wie Kritik am Ausschluss von psychischer Alterität geübt wird, macht aber auch Unterschiede in Hinblick auf Betroffenheit, Teilhabe und Argumentationen deutlich.

## Abstract (en)

This article focuses on performative articulations of critiques of psychiatry, with two forms of demonstration in particular: First, the Mad Pride Parades, which have been held in various German cities since 2013, and second actions by the “Blaue Karawane” in Bremen, a movement that emerged in the 1980s in the wake of the dissolution of a psychiatric clinic. Although they are situated in different temporal and local contexts, both rely on forms of street protest to question the demarcation between ‘normal’ and ‘mad’ and to promote the equal recognition of mental alterity. A detailed examination of the forms of action highlights the importance of carnivalesque celebration, provocation and spectacle for both forms of psychiatric critique. We argue that these forms allow experiencing this critique and, at the same time, make drafts of a different—better—society tangible. To elaborate on these aspects, we draw on queer and gender theoretical considerations as well as on approaches from performance studies. Looking at Mad Pride parades and the Blaue Karawane in their respective contexts reveals similarities in the way in which critique of the exclusion of mental alterity is articulated, but also highlights differences in terms of affectedness, participation and argumentation.

„Tanzt Barrieren weg! Hüpft aus den Schubladen! Scheißt auf Diagnosen! Wir wollen eine Gesellschaft, die bereit ist, Barrieren abzubauen, statt Menschen als ‚krank‘, ‚gestört‘ und ‚nicht normal‘ auszusortieren!“ (Behindert und verrückt feiern – Pride Parade Berlin 2013a)

Mit diesen Worten riefen Aktivist*innen^1^ des „Arbeitskreises mit_ohne Behinderung“ (AK moB) und des „AK Psychiatriekritik“ 2013 zur ersten „Behindert und verrückt feiern – Pride Parade Berlin“ auf. In diesem wie auch den folgenden Jahren kamen bis zu 2.000 Menschen zusammen, um sich für eine inklusivere Gesellschaft und den Abbau von Diskriminierung einzusetzen. Mit dem Slogan „So, wie wir sind, sind wir richtig!“ zogen die Teilnehmer*innen der Parade durch die Straßen, traten für die Anerkennung psychiatrieerfahrener und behinderter Menschen ein und wandten sich gegen psychiatrische Praktiken der Klassifizierung und Medikalisierung. Mittlerweile gibt es Mad Pride-Paraden^2^ auch in anderen Städten des deutschsprachigen Raums, etwa in Köln, Wien und Greifswald.

Diese Paraden haben Vorläufer:^3^ Bereits in den 1980er Jahren gingen Aktivist*innen für eine radikale Umstrukturierung des psychiatrischen Versorgungssystems auf die Straße. Die Bremer Initiative „Blaue Karawane“ organisierte 1985 eine Rundreise mit ehemaligen Patient*innen, Psychiater*innen und Künstler*innen zu psychiatrischen Großkliniken in der Bundesrepublik. Sie prangerte die Bedingungen in den Kliniken an und forderte deren Auflösung sowie neue Formen der Langzeitbetreuung. Öffentlich kritisiert wurde die gesellschaftliche Stigmatisierung und Ausgrenzung von Menschen mit psychiatrischen Diagnosen und geistigen Behinderungen. Auf die erste Karawane folgten drei weitere, die auf unterschiedlichen Reiserouten neben der Psychiatrie auch andere Formen gesellschaftlicher Ausgrenzung thematisierten.

Mad Pride-Paraden und die Blaue Karawane sind Momente, in denen Kritik an der Psychiatrie sowie an gesellschaftlichen Normalisierungs- wie Normierungsprozessen mittels öffentlicher Umzüge in den Stadtraum getragen wird. Das „Verrückte“ und „Nicht-Normale“, das lange Zeit hinter Anstaltsmauern verborgen war, sowie alle Formen von Behinderung werden bei diesen Umzügen demonstrativ öffentlich und damit als Teil der Gesellschaft sichtbar und – wichtiger noch – als Effekte gesellschaftlicher Normierung verhandelbar gemacht. Zugleich führen diese Performances Visionen einer anderen Gesellschaft auf und vor.

Öffentlicher Straßenprotest, auch in der spezifischen Form einer Parade, findet dort statt, wo das Eingreifen in Ordnungen – des Moralischen, des Politischen, des Juridischen – den Veranstaltenden am sinnvollsten erscheint. Dort, wo Bezüge zu bestimmten Orten und anderen Personen hergestellt werden können, werden Aussagen und Forderungen der Demonstration für Zuschauende lesbar und erfahren Aufmerksamkeit. Zugleich sind die Aufzüge performative Momente, bei denen nicht nur Anliegen präsentiert werden, sondern auch das „Noch-Nicht“ (Bloch 2016 [1935]) einer möglichen – anderen, „besseren“ – Gesellschaft aufscheint. Aus dieser Perspektive gehören solche Aktionen zu den stets umstrittenen Politiken, in denen gesellschaftliches Zusammenleben verhandelt wird (Bodden 2022: 400). Demonstrationen schaffen im „Zusammengeworfensein“ (*throwntogetherness*, Massey 2015: 141f.) Raum für eigene Anliegen.

Beide Demonstrationen stehen für die Forderung nach Sichtbarkeit und den Anspruch auf Anerkennung psychischer wie körperlicher Alterität. Die Mad Pride-Paraden wie auch die Blaue Karawane artikulieren wie alle Demonstrationen öffentlich Forderungen und tragen damit auch zur Verständigung der Organisierenden und Teilnehmenden bei (Schönberger & Sutter 2009). Zugleich stellen sie das Diverse, Bunte, „Andere“ der normalisierten Mehrheitsgesellschaft provokativ zur Schau. Anklänge an karnevaleske Veranstaltungen und theatrale Aktionen werden sichtbar (vgl. Denk & Waibel 2009: 72ff.). So kann die Analyse dieser Demonstrationen die Arbeit an der Multiplizität (Bodden 2022) deutlich machen, die wie alle sozialen Kämpfe in einem machtvoll strukturierten Raum stattfindet (Massey 2015; Schuster 2016).

Im Folgenden wollen wir diesen beiden Aspekten – der Ausgestaltung der öffentlichen Intervention und der performativen Aufführung gesellschaftlicher Visionen – genauer nachgehen, dabei Unterschiede und Gemeinsamkeiten der beiden Aktionen in den Blick nehmen und die Spezifik der jeweiligen „Arbeit an der Multiplizität“ zeigen. Wir stützen uns dabei auf Archivmaterialien, Selbstdarstellungen, Interviews, Medienberichte und zeitgenössische wie aktuelle Literatur, wie im weiteren Verlauf der Argumentation deutlich werden wird.^4^ Mit Fokus auf der Artikulation von Psychiatriekritik fragen wir danach, welche Rhetoriken und Imaginationen mit diesen Protestformen mobilisiert, wie Psychiatrie und psychiatrische Gewalt inszeniert, dabei Normierung und Normalisierung hinterfragt und mit möglichen Zukünften konfrontiert werden.

Die Normierungen, die in psychiatrischen Praktiken, aber auch im gesellschaftlichen Umgang mit psychischer Alterität hervorgebracht werden, sind intersektional geschlechtlich strukturiert (Hölling 2000: 79). Um die expliziten wie auch impliziten intersektionalen Bezüge auf Geschlecht und Normalität aufzuzeigen, greifen wir auf gender- und queertheoretische Ansätze zurück. Zunächst werden wir jedoch die Blaue Karawane und die Mad Pride-Paraden in ihren jeweiligen Entstehungskontexten und mit ihren gesellschaftspolitischen Bezugspunkten genauer vorstellen. Anhand einzelner Elemente der jeweiligen Inszenierungen diskutieren wir im Anschluss die Rolle karnevalesker und provokativer Aktionen und fragen, wie in solchen Momenten Normierungsprozesse, insbesondere die Unterscheidung normal#verrückt^5^, herausgefordert werden. Abschließend gehen wir auf die gesellschaftlichen Visionen ein, die in diesen Inszenierungen sichtbar werden.

## Blaue Karawane und Mad Pride-Paraden: Psychiatriekritische Demonstrationen im Kontext

In einem ersten Schritt wollen wir die Entstehungsbedingungen, Hintergründe und gesellschaftlichen Bezugspunkte der beiden psychiatriekritischen Aktionen genauer in den Blick nehmen. Dabei geht es uns weder um einen formalen Vergleich noch darum, die beiden Aktionen in eine (lineare) historische Kontinuität zu stellen. Vielmehr betrachten wir die eine Aktion im Licht der anderen, um auf diese Weise sowohl Gemeinsamkeiten wahrnehmen als auch Unterschiede identifizieren zu können. Dafür beschreiben wir die Demonstrationen mit einem weiten Blick für Kontexte und Bezüge sowie gleichzeitig kleinteilig sensibel, mit genauem Blick für ihr spezifisches empirisches Verhaftetsein (Chakkalakal 2021: 135).

### Die Großkliniken auflösen: Bundesweite Protestfahrten der Blauen Karawane

„Die Unheilbaren kommen – Aus dem Asyl in die Stadt.“^6^ Mit diesem Banner präsentiert die Blaue Karawane auf der Titelseite einer Broschüre ihr Vorhaben einer Reise durch bundesdeutsche Städte mit psychiatrischen Großkliniken. Die Reise, so verdeutlicht die Broschüre, soll dem Erfahrungsaustausch und der Bestandsaufnahme zum Stand der Psychiatriereform dienen. Darüber hinaus will sie Raum für Geselligkeit eröffnen mit Musik, Theater sowie gemeinsamem Trinken, Feiern und Tanzen.^7^

Die Karawane erregte Aufmerksamkeit, wie die zahlreichen Medienberichte in Lokalzeitungen über die Aktion verdeutlichen.^8^ Diese zeigen auch, wie sehr Psychiatriekritik zu dieser Zeit ein öffentlich diskutiertes Thema war. Bereits seit den 1970er Jahren wurden die Missstände in Psychiatrien verstärkt medial ins Licht gerückt. Insbesondere im Kontext der 68er-Bewegung entstand ein gesellschaftliches Klima, in dem sowohl der Umgang mit Minderheiten generell als auch antipsychiatrische Positionen diskutiert wurden. Diese Aufmerksamkeit wurde durch die vom Deutschen Bundestag eingesetzte Psychiatrie-Enquete-Kommission verstärkt, die 1975 ihren Bericht mit entsprechenden Handlungsempfehlungen vorgelegt hatte (Reumschüssel-Wienert 2021: 52). Insbesondere das Prinzip der Anstalt und die langfristige Unterbringung von Patient*innen wurden zunehmend und grundsätzlich infrage gestellt (Rudloff et al. 2022: 4). So forderte die Deutsche Gesellschaft für Soziale Psychiatrie (DGSP) die Auflösung der Großkliniken und die Entwicklung ambulanter Alternativen, die die Isolation von Menschen mit psychiatrischen Diagnosen und geistigen Behinderungen beenden sollten.^9^

Im Rahmen der Bremer Psychiatriereform, die durch das Bundesmodellprogramm 1979/80 angestoßen wurde, wurde beschlossen, die Klinik Kloster Blankenburg bei Oldenburg aufzulösen (Kruckenberg 1991: 91ff.). Bislang waren die Patient*innen durchschnittlich 16 Jahre lang in der Klinik Blankenburg untergebracht (Blaue Karawane o.J.). Viele Bewohner*innen hatten zuvor in Bremen gewohnt, jedoch über die Jahre den Bezug zu der etwa 30 Kilometer entfernten Stadt verloren. Im Prozess der Auflösung der Klinik wurde in Blankenburg eine Enthospitalisierungsstation eingerichtet. Dort sollten die Bewohner*innen nicht nur den Weg in ein Leben außerhalb der Klink finden, sondern auch Zugänge zu sich selbst und ihrem eigenen Leben zurückerlangen (Möller 1991: 46). Auf der Station wurde versucht, die hierarchischen Strukturen der Psychiatrie aufzulösen, indem Räume des Austauschs und Gesprächs geschaffen wurden, in denen zum Beispiel Psychiater*innen und Patient*innen gemeinsam durch die Patient*innenakten gingen und Medikationen verhandelten (Pramann 1990: 41).

Im Zuge dieses Prozesses entstand auch die Initiative Blaue Karawane, die maßgeblich von Mitarbeitenden der Enthospitalisierungsstation getragen wurde. Sie beriefen sich auf einen umfassenden Begriff von Betroffenheit, der über die individuelle Erfahrung der Insassen von psychiatrischen Kliniken hinausging. In dem Film *Das Narrenschiff* (Blumental 2009), der die vierte Blaue Karawane begleitet, befragt einer der Filmemacher*innen den Psychiater Klaus Pramann, der in die Auflösung der Klinik Blankenburg eingebunden und maßgeblich an der Planung der Blauen Karawane beteiligt war, danach, wie viele „Betroffene“ an der Reise teilnehmen. Pramann antwortete: „Ja, hier frage ich mich, ob ich betroffen bin. Da bringen Sie uns echt in Schwierigkeiten, weil es sich da auflöst“ (ebd.: 00:14:00). Wie Pramann verstanden auch andere die Frage einer besseren Versorgung von Menschen mit psychiatrischen Diagnosen und geistigen Behinderungen nicht als individuelles Problem einer Minderheit, sondern als gesamtgesellschaftlichen Auftrag. „Irren ist menschlich“, insofern sind alle betroffen, wie es Klaus Dörner, eine der zentralen psychiatriekritischen Stimmen, formulierte (Dörner & Plog 1978). Diese Vorstellung von Betroffenheit machte das gemeinsame Agieren von Mitarbeitenden und Patient*innen möglich.

Zudem war die Blaue Karawane maßgeblich von der italienischen Reformbewegung inspiriert, die für eine grundlegende Umwälzung der psychiatrischen Versorgung eintrat (Beyer 2022: 161). Die Reformbewegungen in Deutschland folgten dem italienischen Ansatz allerdings nicht in allen Aspekten. So forderte etwa die DGSP keinen „psychiatrischen Kahlschlag“, sondern setzte auf Modernisierung und die dezentrale Betreuung von als chronisch kategorisierten Personen. Die Auflösung der Anstalten fand insofern eher punktuell statt, während Modernisierungsnarrative weiterhin die Deutungshoheit behielten (Beyer 2022: 171f.). Die Blaue Karawane ging hier weiter: Sie trat für eine Auflösung ohne heimartige Unterbringung ein. Sie stand insofern in einem Spannungsverhältnis zu den Reformplänen in Westdeutschland, indem sie einen radikaleren Ansatz vertrat. Diese Position geriet auch in Konflikt mit der Heimleitung von Blankenburg, die die Auflösung letztlich nicht nur durch ambulante Alternativen umsetzen wollte, sondern auch neue, heimähnliche Unterbringungen vorsah (Interview Klaus Pramann, 17.11.2023).

Um die eigene Position zu stärken, stellten die Aktivist*innen der Blauen Karawane Verbindungen zur italienischen Bewegung her. Eine Gruppe von Blankenburger Patient*innen und Mitarbeitenden reiste 1983 an die ehemalige Wirkungsstätte Franco Basaglias in Triest:^10^ Sie war begeistert von den Erfahrungen in der bereits aufgelösten Triester Klinik und gleichzeitig resigniert über den Verlauf der Reformen in Blankenburg. Im Anschluss an den Triest-Besuch beschloss die Gruppe, eine Rundreise durch deutsche Psychiatrien zu unternehmen und für diesen anderen Weg zu werben (Blaue Karawane o.J.).

Die erste Karawane startete im August 1985 in Triest und wurde von unterschiedlichen künstlerischen Aktionen, Theater und Musik sowie von zwei großen Figuren begleitet: dem Pferd Marco Cavallo, das zum Symbol der Befreiung der Anstalt in Triest geworden war,^11^ und einer Nachbildung der Bremer Stadtmusikanten. An der Karawane nahmen etwa 60 Personen teil: ehemalige Bewohner*innen von Blankenburg, Mitarbeitende der Klinik, Künstler*innen und Musiker*innen. Auch Aktivist*innen aus der italienischen Bewegung reisten mit. Die Karawane fuhr im Sommer 1985 durch die Bundesrepublik und besuchte neun psychiatrische Krankenhäuser.^12^ Bei deren jeweiliger Leitung forderten die Teilnehmenden Besuche bei Patient*innen ein, auf den Marktplätzen der einzelnen Städte spielten sie Theater und versuchten, mit Vertreter*innen der Politik zu diskutieren. Die Karawane argumentierte für die Auflösung der Großkliniken nicht aus therapeutischen Gründen, sondern berief sich vielmehr auf Menschenrechte und die Unantastbarkeit der Menschenwürde (Interview Klaus Pramann, 07.11.2023). Den Abschluss der Karawane bildete der Kongress „Gesellschaft ohne Irrenhaus“ des Réseau-Alternative à la Psychiatrie (Blaue Karawane o.J.) – eines international agierenden Zusammenschlusses psychiatriekritischer Initiativen und Einzelpersonen.^13^ Auf die erste Karawane folgten drei weitere, die jeweils unterschiedliche Formen gesellschaftlicher Ausgrenzung und Normierung in den Fokus rückten.^14^

### Behindert und verrückt feiern: Die Aneignung stigmatisierender Zuschreibungen durch die Mad Pride-Bewegung

Anliegen wie Hintergrund der Mad Pride-Paraden unterscheiden sich in vielerlei Hinsicht von der Blauen Karawane. Zwar zielen diese Paraden auch auf gesellschaftliche Anerkennung und einen veränderten Umgang mit psychischer Alterität. Doch wie der Aufruf zur ersten Mad & Disability Pride-Parade in Berlin deutlich macht, rufen hier „Betroffene“ selbst dazu auf, normierende gesellschaftliche Erwartungen zu unterlaufen: „Küsst den Wahnsinn wach, liebt Krummbeine und Spasmus, begehrt Krücken und Katheter! Malt Eurer Scham Pink und Glitzer auf die Wange und lasst sie laufen!“ (Behindert und verrückt feiern – Pride Parade Berlin 2013a). Sprachlich vorweggenommen wird der Spaß am gemeinsamen Feiern auf der Straße. Im Mittelpunkt des Aufrufs steht dabei die Aneignung und das Umdrehen von stigmatisierenden Zuschreibungen. Adressiert werden nicht „Betroffene“ von Behinderung und psychiatrischen Diagnosen, sondern Personen, die selbstbewusst nicht normgerechte Verhaltensweisen und Körper in die Öffentlichkeit tragen und ihre Sehnsüchte, Wünsche und Rechte gegenüber einer Mehrheitsgesellschaft einklagen, die ihrem eigenen Leben Barrieren in den Weg stellt.

Die Mad Pride-Paraden zielen darauf, die Abweichung von der Norm psychischer wie körperlicher Gesundheit positiv zu besetzen, indem gesellschaftliche Abwertung in Stolz verwandelt wird (Tietz 2016: 196). Mit dieser Strategie schließen sie an queere Bewegungen an, die seit 1970 zunächst in den USA in Erinnerung an die Stonewall Riots von 1969 (Bravmann 2003) und den Widerstand gegen staatliche Repression queeren Lebens durch eine Vielzahl von Städten ziehen. Dieses Prinzip der Selbstermächtigung fand in den Mad Pride-Paraden erneut Widerhall – erstmals 1993 im kanadischen Toronto unter dem Titel „Psychiatric Survivor Pride Day“ (Pfahl & Köbsell 2016; Beresford & Russo 2021; s. auch Toronto Mad Pride 2016). Seitdem finden in verschiedenen nordamerikanischen Großstädten Pride-Paraden von Psychiatrieerfahrenen, Behinderten sowie queeren, trans und inter Personen statt. Schließlich wurde diese Protestform im europäischen Raum übernommen und auch hier Paraden mit dem doppelten Ziel des eigenen Empowerments und des Protests gegen Ausgrenzung, Stigmatisierung und Diskriminierung ausgerichtet.

Gleichzeitig führen die deutschen Mad Pride-Paraden auch lokale Protestformen fort: In den 1970er Jahren waren an verschiedenen Orten im deutschsprachigen Raum sogenannte Krüppelgruppen, Krüppelfrauengruppen oder Krüppellesbengruppen entstanden (Fuchs 2022; Pfahl & Köbsel 2016). Zur gleichen Zeit begannen sich auch psychiatriekritische Initiativen zu formieren, wobei die Irren-Offensive, der Bundesverband Psychiatrie-Erfahrener, Stimmenhör*erinnen, Trialoge und Weglaufhäuser zunächst ohne Bezug zur Behindertenbewegung agierten (Lüthi 2022). Anders als bei der Krüppelbewegung unterscheiden sich die psychiatriekritischen Initiativen nicht durch ihren Bezug auf Geschlechterverhältnisse und Sexualitätsregime, sondern hinsichtlich ihrer Ansatzpunkte für eine Kritik psychiatrischer Praxis.

Die Mad Pride-Paraden sind schließlich auch im Kontext von akademischen Versuchen zu sehen, psychische Diversität jenseits psychiatrischer und medikalisierter Zugänge zu analysieren. Diese dichte Verzahnung von aktivistischen und wissenschaftlichen Auseinandersetzungen, wie sie seit den 1970er Jahren für verschiedene soziale Bewegungen charakteristisch ist, artikuliert sich im Feld der Psychiatriekritik in den Mad Studies^15^. Im deutsprachigen Raum melden sich seit den 1990er Jahren psychiatrieerfahrene Wissenschaftler*innen zu Wort, die sich kritisch mit psychiatrischer Praxis und deren Institutionen auseinandersetzen (Hölling 2000; Russo 2012; von Trotha 1993). Ausgehend von antipsychiatrisch geprägten Betroffenenperspektiven (Beresford & Russo 2021; LeFrançois et al. 2012) versuchen Ansätze der Mad Studies, Erfahrungswissen als Gegennarrativ zu biomedizinischen Expertisen zu etablieren (Lüthi 2022; Tang 2021: 268). Sie weisen den Anspruch von Psychiatrie und Biomedizin auf einen vermeintlich richtigen Zugriff auf psychische Alterität zurück (Beresford 2020: 1337; Spandler & Barker 2016). Ausgehend von der selbstbewussten Selbstbezeichnung als Mad oder verRückt (Lüthi 2019, 2022) steht die Auseinandersetzung mit psychiatrischen Wissensordnungen sowie psychiatriekritischen Praktiken im Zentrum dieses emergenten Forschungsfeldes (siehe unter anderem Ingram 2016; LeFrançois et al. 2016). Psychische Alterität und Behinderung werden als gesellschaftliche Relation verstanden, die Alltag und Gesellschaft machtvoll strukturiert, indem spezifische menschliche Fähigkeiten vorausgesetzt werden, deren Nichtvorhandensein entsprechend als Mangel gilt (Pfahl & Köbsell 2016: 71).

Wie Helen Spandler und Meg-John Barker (2016) zeigen, bestehen mit dieser macht- und normalisierungskritischen Ausrichtung der Wissensproduktion auch enge Verbindungen zu den Queer Studies – nicht zuletzt aufgrund der beiden Wissensfeldern gemeinsamen grundsätzlichen Infragestellung von Bio- und Psy-Wissenschaften (vgl. auch McRuer 2023; Laufenberg 2019: 332). Allerdings ist die positive Besetzung des Begriffs *mad* umstritten. Gegen seine Umdeutung wird ins Feld geführt, dass *mad* angesichts möglicher retraumatisierender Effekte durch das Aufrufen psychischer Erfahrungen sowie aufgrund eines verstörenden Erlebens psychischer Alterität nur schwer positiv angeeignet beziehungsweise umcodiert werden kann (Beresford 2020).

Mit der umstrittenen Neubesetzung der Begriffe *mad* oder verrückt bewegen sich die Mad Studies damit – wie auch die Mad Pride-Bewegung – in einem Spannungsfeld zwischen der Behauptung einer spezifischen, je individuellen Position jenseits der Norm einerseits und dem Anspruch nach Anerkennung als Gleiche, also als Teil einer erweiterten Vorstellung des Normalen andererseits. In Korrespondenz damit wird Verrücktsein als Erfahrungsweise und Subjektposition politisiert (Spandler & Barker 2016). Deutlich wird das etwa im Aufruf zur Kölner Mad Pride von 2021:

„Erlebt Ihr uns als irritierend, wenn wir sind, wie wir sind? Meint Ihr unser Alltag wäre leidvoll? Dann ist das nicht unser Problem. Wir sind lustvoll und zugewandt, verlieben uns, haben Beziehungen und bekommen Kinder. Wir essen, schlafen, gehen zum Arzt, manche arbeiten, genießen, lachen und weinen – und feiern bei der MAD PRIDE uns selbst und unser Leben“ (Sommerblut 2021).

Auch hier klingt beides an: Die Aneignung der stigmatisierenden Bezeichnung *mad* und zugleich das Insistieren auf Normalität. Diese doppelte Bewegung macht Vernetzung mit anderen Betroffenen möglich, wobei zugleich davor gewarnt wird, die diversen individuellen Erfahrungen vorschnell in einem „Wir“ aufgehen zu lassen (Lüthi 2022). Es ist ein grundlegendes Prinzip der Pride-Paraden, dass zum Beispiel verrückte und behinderte Aktivist*innen in einem Bündnis zusammenarbeiten, um sich, wie auf der Webseite zu lesen ist, der Pathologisierung und der gemeinsamen Erfahrung entgegenzustellen; der Erfahrung, „dass die Mehrheitsgesellschaft sie durch ‚Therapien‘ an ihre Normen anpassen will“, denn: „Wer nicht ‚funktioniert‘, wird ausgeschlossen bzw. weggesperrt.“ (Ebd.) Auf der Webseite und in Redebeiträgen werden zudem trans und inter Menschen einbezogen, die ebenfalls durch Gesetze (z. B. Transsexuellengesetz) oder durch diagnostische Verfahren pathologisiert werden (Drebes & Reher 2021).^16^

Die Bündnispolitiken und die entsprechenden Aufrufe machen den widersprüchlichen Status der Grenze von normal#verrückt deutlich: Stigmatisierung und Ausgrenzung psychischer wie körperlicher Alterität bestehen fort, auch wenn die Grenzen des „Normalen“ deutlich aufgeweicht wurden, wie die Gegenüberstellung der beiden Aktionsformen zeigt.

### Psychiatriekritischer Protest in Relation

In der relationalen Betrachtung der beiden Ereignisse werden die Unterschiede deutlich, die sich aus der jeweiligen Situierung der beiden Aktionen ergeben: Formen der Subjektivierung und Möglichkeiten zu Allianzbildungen haben sich in den letzten Jahrzehnten verändert. Während die Blaue Karawane einem Moment entsprang, an dem Kritik an der Psychiatrie ein zentrales gesellschaftliches Thema war, sind die Mad Pride-Paraden eher eine Aktionsform, die wenig mediale Aufmerksamkeit erhält. Sie finden ihren Resonanzraum vornehmlich in dem alternativen Milieu, in dem sie situiert sind. Auch der gesellschaftliche Umgang mit psychischer Alterität hat sich verändert. Zwar wird Psychiatrieerfahrung nach wie vor stigmatisiert, doch alle möglichen Formen der Psychotherapie sind weitgehend alltäglich geworden. Die Enthospitalisierungsbewegung der 1970er und 1980er Jahre war an der Durchsetzung eines flexiblen Normalismus beteiligt, wie er für gegenwärtige Gesellschaften kennzeichnend ist (Jäger/Jäger 2007: 61–69): Starre Vorstellungen eines klar umrissenen „Normalen“ wurden ebenso wie klare Grenzziehungen zwischen dem Normalen und dem „Verrückten“ aufgeweicht. Doch noch immer wird keinesfalls jedes Verhalten und jede Abweichung toleriert. „Wie eng die Grenzen gesetzt sind, wie weit der Bogen überspannt werden kann, ohne Strafaktionen befürchten zu müssen, ist offenbar abhängig vom jeweiligen Handlungsfeld und den mit ihm verbundenen Diskursen und Operationen“, schreibt Anne Waldschmidt (1998: 4). Psychiatriereformen und die Entstehung ambulanter Versorgungsstrukturen der Gemeindepsychiatrie führten dazu, dass die Präsenz psychischer und körperlicher Alterität im Straßenbild alltäglicher geworden ist. Personen mit körperlichen und geistigen Einschränkungen leben genauso wenig wie „Verrückte“ ausgegrenzt in Kliniken am Stadtrand, sondern haben, wenn auch noch immer mit vielen Hindernissen konfrontiert, teil an der Gesellschaft. Dennoch weisen Kritiken am Konzept des flexiblen Normalismus darauf hin, dass insbesondere Menschen mit Behinderungen und psychiatrischen Diagnosen mit Erwartungen der Anpassung und der Selbstnormalisierung konfrontiert sind (ebd.: 19).

Für gesellschaftskritische soziale Bewegungen sind Fragen nach dem Gehörtwerden von Kritik und die Anerkennung von Sprecher*innenschaft zentral (Yates 2021). Damit Kritik in der Öffentlichkeit wahrgenommen wird, müssen die Botschaften mit Wahrnehmungs- und Argumentationsweisen räsonieren, die in machtvoll strukturierten Diskursen ausgehandelt werden (Spivak 1988). Dies gilt auch für die Psychiatriekritik, wie sie die Blaue Karawane und die Mad Pride-Paraden vortragen. Beide sind in den politischen Bewegungen und Kämpfen ihrer Zeit situiert, werden von Selbstverständnissen und Vorstellungen ihrer jeweiligen Gegenwarten getragen, was auch dazu führt, dass Sprecher*innenschaften und Deutungsmacht unterschiedlich organisiert sind. Während die Mad Pride in erster Linie eine Bewegung von Psychiatrieerfahrenen ist, ging die Blaue Karawane vornehmlich aus dem Engagement der Mitarbeiter*innen der Enthospitalisierungsstation der Klinik hervor. In der Mad Pride-Bewegung haben sich offensichtlich weitgehend solche Positionen durchgesetzt, die die Anliegen von Betroffenenbewegungen einerseits und Bewegungen von Professionellen andererseits für inkompatibel halten (von Trotha 2001; Russo & Hölling 2022). Damit wird einerseits Erfahrung verabsolutiert und andererseits gegen Kritik immunisiert, indem gegen Praktiken der Objektivierung auf der Situiertheit von Wissen beharrt wird.

Vor dem Hintergrund der Entwicklung von Normalisierungspolitiken wird auch deutlich, wie die beiden Bewegungen die Divergenz von *normal* und *verrückt* unterschiedlich formulieren. Die Relationierung macht das Aufweichen der Grenzen sichtbar, wie wir im Folgenden noch genauer zeigen werden, wenn wir den jeweiligen Formen nachgehen, mit denen die Blaue Karawane und die Mad Pride-Paraden Kritik an Normierungs- und Normalisierungsprozessen übten und üben.

## Normal#verrückte Verhältnisse zum Tanzen bringen: Parade und Karawane als Protestform

Werden die Formen des Protests in den Blick genommen, fällt zunächst der bunte, fröhlich-festive Charakter beider Umzüge auf. Solche Auftritte zählen seit den 1960er und 1970er Jahren zum Repertoire des Straßenprotests. Hierzu passt auch das Anliegen, Demonstrationen zu nutzen, um öffentlich – eben demonstrativ – gegen Stigmatisierung und die Kategorisierung normal#verrückt zu intervenieren. Die Mad Pride-Paraden ähneln in ihrer Form gängigen Demonstrationen. Paraden sind, wie Demonstrationen, zeitlich begrenzte Ereignisse, bei denen unterschiedliche Menschen und Gruppen kurzfristig zusammenkommen. Gleichzeitig wird durch die Organisationsteams während der Vorbereitungszeit Kontinuität gewährleistet, die nach innen vernetzt und nach außen Wiedererkennung schafft (Schönberger & Sutter 2009). Auch die Rede- und Musikbeiträge zum Abschluss von Demonstrationen tragen dazu bei, das Netzwerk aus Betroffenen, Unterstützer*innen und Gleichgesinnten zu festigen, Anlaufstellen und Vereine sichtbar zu machen sowie über das kurzfristige Agieren auf der Straße hinaus zu wirken.

In ihrer aktuellen Artikulation sind die Mad Pride-Paraden lokale Ereignisse, die sich die räumliche Struktur der jeweiligen Orte zunutze machen. Die erste Berliner Mad Pride zog 2013, begleitet von Lautsprecherwagen und Transparenten, durch die Bezirke Neukölln und Kreuzberg. Bei einem Zwischenhalt wie auch beim abschließenden Bühnenprogramm mit Musik und Reden von beteiligten Organisationen und Personen werden unterschiedliche Positionen sichtbar.^17^ Wie Beteiligte selbst betonen (AK Psychiatriekritik 2015: 311f.), verzahnt sich der Wunsch, sich selbst zu feiern, mit dem Anliegen, über die eigenen Lebensbedingungen aufzuklären und Kritik zu üben: Redebeiträge, Transparente und Aktionen sollen „psychiatriekritische Inhalte an zahlreiche Menschen“ vermitteln (ebd.: 311). Die Route verbindet, wie auch in den folgenden Jahren, Orte miteinander, die bereits für das „andere“ Berlin stehen, für Protest und Widerstand sowie für das Erproben neuer Lebensweisen (Lang 1998; Schuster 2016). Doch die Paraden finden zu einem Zeitpunkt statt, an dem Psychiatriekritik kaum noch öffentlich diskutiert wird. Da zudem nahezu täglich Demonstrationszüge durch Berlin wie auch andere bundesrepublikanische Städte ziehen, findet eine einzelne Veranstaltung kaum noch Widerhall – anders als noch bei der Blauen Karawane der Fall.

Auch die Blaue Karawane war zunächst ein flüchtiges Zusammentreffen, insgesamt jedoch langfristiger angelegt als die Pride-Paraden. Immerhin reisten die Teilnehmer*innen über einen Zeitraum von mehreren Wochen gemeinsam durchs Land. Eine feste Gruppe, die oft während der ganzen Route zusammenblieb, traf vor Ort dann mit Bewohner*innen der jeweiligen psychiatrischen Anstalten oder Städte zusammen.

Die Selbstbeschreibung als Karawane eröffnet dabei einen Assoziationsraum zu anderen Formen des kollektiven Reisens. Sie erinnert beispielsweise an Pilgerreisen oder Wallfahrten, bei denen das gemeinsame Unterwegssein und die dadurch entstehenden Gemeinschaftsgefühle als wesentlich für die transzendentale Erfahrung verstanden werden (Röhm 1985: 83f.). Auch bei der Blauen Karawane verließen die Teilnehmenden ihre Heimatorte in der Hoffnung, unterwegs Neues, ihren Alltag Aufrüttelndes zu finden und gemeinsam verändernde Erfahrungen zu machen. So ist in einer Broschüre zur ersten Blauen Karawane zu lesen:

Gespräch mit Kurt:

Warum wollen Sie die Reise mitmachen?

Immer in Blankenburg dieselben Leute grüßen, das fällt mir schwer! Ich will andere Leute sehen.^18^

Die Gruppe bereiste allerdings keine Orte der Erweckung oder der Läuterung, wie es bei Wallfahrten der Fall ist. Im Gegensatz dazu besuchte sie Orte, an denen die gesellschaftspolitische Kritik am Umgang mit psychischer Alterität und Psychiatriekritik sichtbar wurde. Insofern erinnert die Protestform der Blauen Karawane als organisierte Bewegung einer Gruppe mit einem politischen Anliegen auch an die Ostermärsche der Friedensbewegung, die – inspiriert durch Proteste in England – seit den 1960er Jahren in der Bundesrepublik gegen die atomare Aufrüstung demonstrieren (Aehnelt 1982: 21f.).

Bei der Blauen Karawane bildeten unterschiedliche Formate des Austauschs die wichtigen Momente für das Weiterentwickeln und Festigen gesellschaftspolitischer Positionen, aber auch der eigenen Rolle als Expert*innen für Psychiatriekritik.^19^ Zudem bot das Straßentheater ein Format für Begegnungen im städtischen Raum. Dabei spielten Parallelen und Bezüge zu Formen des Straßentheaters, die in den 1968er Jahren entstanden waren, eine Rolle. Auf diese Weise sollte das Publikum zur Teilhabe an einer grundlegenden Veränderung der Gesellschaft motiviert werden (Kraus 2007: 94). Ein zentrales Mittel dieser Straßentheateraufführungen war die bewusste Provokation. Durch Zuspitzung, Übertreibung und emotionalisierende Effekte sollten die Zuschauer*innen in das eigene Anliegen involviert werden.

Bei den Aktionen der Mad Pride-Paraden wie auch bei der Blauen Karawane sind Irritation und Provokation vor allem visuell erfahrbar. Es geht um ein Spektakel im Sinne einer aufsehenerregenden, aufrüttelnden, aber auch informierenden Aufführung. „Wüna“, das „Wüstennarrenschiff“ in der Anmutung eines überlebensgroßen blauen Kamels, das die Reisegruppe seit der zweiten Blauen Karawane 1994 begleitet, überragt die Menschengruppe und stellt eine visuelle Irritation dar. Mateng Pollkläsener, der mit der Theatergruppe *theatre du pain* (o. J.) die Blaue Karawane begleitete, erzählt über die Reise mit Wüna:

„[D]a fährst du über Land, über Wiesen, du siehst auf einmal am Horizont, ähm … nicht am Horizont, irgendwann mal zwischen den Feldern, so ein blaues Kamel über die Elbe schippern. Das war ein echter Eye-Catcher – ich glaube für alle Beteiligten: Kühe“ (Interview 08.04.2024).

Das Gefährt wurde an die jeweiligen Gegebenheiten angepasst: Auf dem Wasser ist das Kamel ein Schiff, an Land steht es auf Rollen. Überall zieht es mit seiner Größe und seiner expressiven Gestalt Aufmerksamkeit auf sich. Auch das *Mindener Tageblatt* beschreibt die Blaue Karawane als „schockierend-faszinierenden Zug“ (Jäger 1994). Wüna – halb Kamel, halb Schiff oder Wagen – steht, so seine Konstrukteur*innen, für das Erkunden von Weite und das Überschreiten von Grenzen. Die Provokationen gingen jedoch über das Spektakel hinaus, wie wir im Folgenden anhand einzelner Aktionen zeigen werden.

## Karnevaleske Kritik und Provokation: Mit der Kettensäge über den Marktplatz

Die Blaue Karawane wurde von künstlerischen Aktionen begleitet. Dafür war unter anderem die bereits oben erwähnte Theatergruppe *theatre du pain* (o. J.) verantwortlich. Klaus Pramann erinnert sich etwa im Interview, wie die Gruppe bei ihrem Besuch in Herborn 1985 die Grenze der Klinik, die sie nicht reinlassen wollte, durch Gummibärchen und Thunfisch symbolisch nachzog (Interview 17.11.2023). Die provokant-spielerischen Aktionen hatten das Ziel, gesellschaftspolitische Diskussionen zu eröffnen, indem sie die Grenzen des Etablierten überschritten.

Besonders deutlich wird der Charakter des provokativen Happenings bei einer Aktion des *theatre du pain* während der zweiten Blauen Karawane, die im Film *Man müsste ne Klatsche haben, wa?* (Besuden 1994) festgehalten wurde. Dort wird in einer Szene ein Streit zwischen zwei Männern nachgestellt, bei dem ein Schauspieler mit einer laufenden Kettensäge durch eine auf einem Marktplatz versammelte Menschenmenge läuft. Eine Stimme aus dem Off kommentiert: „Obwohl diese Straßentheaterszene nur gefährlich aussieht, war für viele Zuschauer die Grenze erreicht. Tatsächlich mitlachen konnten die meisten allerdings erst, als sie genau sahen, dass keine Kette auf der Säge war“ (ebd.: 00:31:30).

Die Theatergruppe spielt mit der Vorstellung, Menschen mit psychiatrischen Diagnosen seien gefährlich und unberechenbar. Weil dies mit der Erwartung des Publikums räsoniert, konnten die Zuschauer*innen erst lachen, als die Kettensäge als ungefährlich enttarnt war. Die Provokation gibt die Vorurteile der Zuschauenden der Lächerlichkeit preis. Es ist kein Zufall, dass es eine männlich zu lesende Person ist, die mit der Kettensäge die Menge in Schrecken versetzt. Die Darstellung referiert auf das Bild eines männlichen, gewalttätigen und verrückten Subjekts (Van Veen et al. 2018). Gerade Männlichkeit, traditionell verbunden mit körperlicher – auch unkontrollierbarer – Stärke, kann zur Rechtfertigung von in der Psychiatrie eingesetzten Zwangspraktiken beitragen. Indes gibt es keine wissenschaftliche Evidenz dafür, dass Männer mit psychiatrischen Diagnosen eher gewalttätig werden als andere Männer; ganz im Gegenteil: Sie werden eher Opfer von Gewalt (ebd.: 243, 247). Die Kettensägenaktion verleiht Handlungsmacht und irritiert das Narrativ vom gefährlichen, männlichen Verrückten. Das Spiel deckt bestehende Vorurteile auf und spiegelt sie den Zuschauenden.

Für das Eröffnen von Räumen jenseits der herrschenden Ordnungen wird auch ein karnevaleskes Spektakel genutzt. Dies wird etwa von Hans-Walter Schmuhl (2022) betont, der von einem Kostümfest 1971 berichtet, das maßgeblich die Reformprozesse im Großkrankenhaus Bethel vorangetrieben habe. Anhand von Dokumenten und Artikeln aus der Patient*innenzeitschrift *Der Drücker* zeigt Schmuhl, wie sich bei der Vorbereitung und Durchführung des Kostümfests durch Musik, Kostümierung und gemeinsames Agieren ein Gemeinschaftsgefühl eingestellt habe, das die hierarchische Struktur der Klinik vorübergehend zumindest kurzfristig aufhob (ebd.: 228).

Die spielerischen, spektakulären und teilweise provozierenden Darstellungen weisen über sich selbst hinaus, indem sie Prozesse individueller Bewusstwerdung sowie gesellschaftlicher Veränderung in Gang zu setzen suchen. Um diesen Moment genauer zu verstehen, lohnt ein Blick in Überlegungen zur Bedeutung des Karnevals. Der Kulturtheoretiker Michail Bachtin (1995) hebt in seiner Untersuchung des mittelalterlichen Karnevals hervor, dass ihm hoffnungsvolle Visionen eingeschrieben seien, die das Potenzial besitzen, Normen zu unterlaufen und eine „zeitweise Befreiung von der herrschenden Wahrheit und bestehenden Gesellschaftsordnung“ (ebd.: 58) hervorzubringen.

## Spektakel als Kritik: *Bed-push*-Aktionen und das Inszenieren psychiatrischer Gewalt

Auch die Aktionen der Mad Pride-Paraden arbeiten mit provokanten Aktionen und karnevalesken Elementen. So etablierte sich bei den Paraden rasch das Mitführen eines Krankenhausbettes. Auch auf der Kölner Mad Pride 2016 stellte das mitgeführte psychiatrische Bett einen theatralen Höhepunkt dar (Superbass 2016). Ein Interviewpartner, der mehrfach an der Organisation von Mad Pride-Paraden beteiligt war, beschreibt die Szene als „richtigen Hingucker“:

„Da liegt ein Mensch im Bett, der wird von Leuten in weißen Kitteln festgehalten. Ja, weil das macht das dann plakativ, was ja eigentlich innerhalb der Psychiatrie stattfindet, was die Leute gar nicht glauben, dass es das vielleicht noch gibt, dass Menschen so behandelt werden“ (Interview 20.12.2021).

Das Krankenhausbett als Symbol psychiatrischer Gewalt wurde in diesen Aktionen also zum Mittelpunkt einer Szene, die Erfahrungen mit Zwangsfixierungen und -medikamentierung aus der psychiatrischen Klinik in die Öffentlichkeit tragen sollte. Der Interviewpartner erzählt, dass die Aktion von der Öffentlichkeit toleriert wurde, sie jedoch von einigen Passant*innen, gerade wenn diese selbst in der Psychiatrie arbeiteten, als Provokation aufgefasst wurde. Auch wenn die Aktion zu Diskussionen geführt habe, betont unser Interviewpartner nachdrücklich ihre Sinnhaftigkeit:

„Also, wenn wir jetzt als Psychiatrieerfahrene ohne Bett durch die Straßen gegangen wären, hätte man uns nicht erkannt. Also das ist ja nicht so in dem Sinne erst mal sichtbar. […] Unsere Barriere ist sichtbar zu machen, weil halt eben die Zwangsbehandlung ist … ja, die ist ja ein großer Teil der Barriere. Dass sozusagen gegen diese Personengruppe der psychisch Kranken Gewalt ausgeübt werden kann und das auch gesetzlich legalisiert ist“ (Interview 20.12.2021).

Diese Erzählung wie auch die Aktion selbst greift zwei Aspekte auf: die tendenzielle Unsichtbarkeit der hinter den Anstaltsmauern ausgeübten psychiatrischen Gewalt und deren Skandalisierung durch eine theatrale Darstellung. Wie Fotos der Aktion zeigen, wurde die Gewalt mit einer großen Spritze, einem weißbekittelten Arzt und Fixierungsutensilien spektakulär in Szene gesetzt – als männlich kodierte psychiatrische Macht über wehrlose Körper. Benjamin Abrams und Peter Gardner (2023) beschreiben, dass Objekte zu Symbolen politischer Bewegungen werden, die ontologische Realitäten zu vermitteln vermögen. Diese Objekte seien oftmals durchdrungen von Macht, weil sie abstrakte Ideen vergegenständlichen und kollektive Wahrheiten in vermeintlich objektive Realitäten verwandeln können, die auch Außenstehende verstehen (ebd.: 5). Die Darstellung einer Zwangsfixierung an einem Bett während der Mad Pride-Parade inszeniert für die Zuschauenden wahrnehmbar psychiatrische Gewalt. Zugleich präsentiert und kritisiert sie die Erfahrung von Zwang in der Psychiatrie als kollektives Wissen (Küpper 2022). Das Bett und die anderen psychiatrischen Utensilien werden dabei zu Symbolen dieser Gewalt. Sie zeigen sich gleichzeitig auch als materielle Realitäten, die etwas verdinglichen und zu beglaubigen scheinen, das für viele Beteiligte wie Zuschauende zur eigenen Erfahrung eines psychiatrischen Klinikaufenthalts gehört.

In den *bed push*-Aktionen wird ein weiterer Aspekt deutlich, den Bachtin (1995) als spezifisch für das Karnevaleske versteht. Karneval sei nicht nur eine künstlerisch-szenische Form, sondern an der Grenze von Kunst und Leben verortet. So schreibt er: „Im Grunde ist er das Leben selbst in einer spielerischen Ausformulierung“ (Bachtin 1995: 55). Dieses spielerische Ausformulieren ermöglicht das Thematisieren gesellschaftspolitischer Anliegen. Auch die Theaterwissenschaftlerin Vicki Ann Cremona betont in ihrer Arbeit über den maltesischen Karneval während der britischen Kolonialherrschaft, dass das im Karneval Gezeigte gerade dadurch seine Ernsthaftigkeit erhält, weil es im sicheren Rahmen des Spiels stattfindet (Cremona 2018: 5). Die spielerischen Unterbrechungen des Alltäglichen im öffentlichen Raum erlauben es, im karnevalesken Spiel Machtverhältnisse aufzudecken und anzuprangern. Auch wenn psychische Alterität, wie oben mit Verweis auf Prozesse der flexiblen Normalisierung angedeutet, alltäglicher geworden ist, findet Gewalt in der Psychiatrie noch immer hinter verschlossenen Mauern statt und wird wenig öffentlich thematisiert. Insofern wundert es nicht, dass die Szene in ihrer Übertreibung und Zuspitzung karnevalesk anmutet und Teil eines Spiels zu sein scheint. In diesem Sinn sprechen die Aktionen des *bed push* von einer – zumindest temporären – Umkehrung von Machtverhältnissen durch Mittel des Spektakels und der Provokation. Personen mit Psychiatrieerfahrung begeben sich spielerisch in die Rolle von Mitarbeitenden psychiatrischer Kliniken, die Fixierungen vornehmen und Spritzen setzen. Sie verlassen dabei die Position des passiven Patient*innensubjekts, das diesen Praktiken ausgeliefert ist. Im Spiel ermächtigen sie sich, in die Rolle derjenigen zu schlüpfen, die in Momenten des Zwangs über andere Körper bestimmen können. Sie stören und unterbrechen die vermeintliche Ordnung der Verhältnisse, erzählen eine Geschichte von sich selbst innerhalb der Institution und machen diese im öffentlichen Raum erfahrbar.

Sowohl die Blaue Karawane als auch die Mad Pride-Paraden irritieren mit ihren performativen Aktionen gesellschaftliche Ordnungen. Sie kritisieren den insitutionellen Umgang mit Menschen mit psychiatrischen Diagnosen und hinterfragen die Unterscheidung von normal und verrückt. Doch diese Kritiken stellen nicht alle gesellschaftlichen Normalitätsvorstellungen auf den Kopf. Auch aus Geschlechterperspektive zeichnet sich ein ambivalentes Bild ab: Während die Blaue Karawane diese eher stabilisiert, beziehen sich die Mad Pride-Paraden mit ihrer Solidarisierung mit trans Positionen auf das Brüchigwerden des binären Geschlechtermodells. Eine darüber hinausgehende Thematisierung der Geschlechterdisparität ist auf den Paraden allerdings nicht zu erkennen. Insofern können die Mad Pride-Paraden als Bewegung verstanden werden, die zwar eine intersektionale Kritik an Normierungs- und Machtverhältnissen befördern will, dabei aber ableistische und psychiatrische Normierungs- und Diskriminierungsstrukturen ins Zentrum stellt, während andere eher im Verborgenen bleiben.

## „Behinderte Revolution, verrückter Aufstand“: Imaginationen, Utopien, Zukünfte

„Ich möchte auch im Leben dieser anderen Seite Raum geben, also der kreativen Seite, die auch Verrücktheit zulässt. Nicht nur in meiner Freizeit, eigentlich möchte ich das auch alltäglich tun können. Das kann ich aber in meinem Beruf nicht, also nicht nur in meinem Beruf, das kann jeder in seinem Beruf nicht. Da muss er funktionieren. Dieser eingrenzende Inhalt von Arbeit ist etwas, das wir infrage stellen wollen und wo wir auch ausbrechen wollen. Wir wollen etwas anderes in unserem Leben auch haben“ (Besuden 1994: 00:32:20).

Klaus Pramann reflektiert in diesem Filmausschnitt über das Potenzial von Verrücktheit als befreiendem Möglichkeitsraum, der erlaubt, aus Alltagszwängen auszubrechen. Statt Verrücktheit an die Ränder der Gesellschaft zu verdrängen, so der Gedanke, könnte das Verrückte als Teil der Gesellschaft dazu beitragen, Normen aufzubrechen und jede*m mehr Freiheit ermöglichen. Der Ausschnitt verdeutlicht darüber hinausgehend auch, dass die betrachteten Kritiken an der Psychiatrie über die jeweilige Gegenwart hinaus auf Zukünftiges verweisen. Sie formulieren dabei Hoffnungen, die auf dem „Noch-Nicht“ (Bloch 2016 [1935]; Muñoz 2009) gründen, das in der Gegenwart eingelagert ist. Rebecca Bryant und Daniel M. Knight (2019) argumentieren unter Bezugnahme auf Ernst Bloch, dass in der Lücke zwischen dem Tatsächlichen und dem Potenziellen Hoffnung entsteht. Sie sei das Resultat ebenjenes Versuchs, ein spezifisches Anderes in die jeweilige Wirklichkeit zu bringen (ebd.: 134) und den Blick für ein *doing otherwise* (Grosz 2011) zu öffnen. Knight und Bryant gehen von bestimmten „timespaces of hope“ (2019: 151) aus, in denen sich diese Potenzialitäten verdichten und in die Gegenwart vordringen können. Die Blaue Karawane und die Mad Pride-Paraden können als solche Rahmen verstanden werden, in denen unterschiedliche Visionen des Zukünftigen in den öffentlichen Raum der jeweiligen Gegenwart gebracht werden. Im Folgenden diskutieren wir einige Momente, bei denen dieses Zukünftige als kollektive Vision formuliert wird.

Im Blauhaus, dem gemeinschaftlichen Wohnprojekt, das aus den Aktivitäten der Blauen Karawane hervorgegangen ist, erzählen in einer Ausstellung Zeitungsausschnitte von einer Aktion der Blauen Karawane bei einem Fest im Bremer Stadtteil Walle 1983: „Freude, Trubel und Heiterkeit standen auf dem Programm. Ein Fest was [sic!] von den Bewohnern der Elisabeth- und Zietenstraße veranstaltet wurde. Auch wir aus Blankenburg, [sic!] konnten der netten Einladung dieser Ortsgemeinde Folge leisten.“^20^ Eine Gruppe von Mitarbeiter*innen und Patient*innen der Klinik baute auf der Straße mit Tischen, Stühlen, Sesseln und Getränken ein Wohnzimmer nach und verkaufte die Patient*innenzeitschrift *Klosterbote*. Ein Banner mit der Aufschrift „Ein Bett in der Anstalt ist keine Wohnung! – Wir wollen wieder in Bremen leben“^21^ war Titel wie Programm der Aktion. In dem Slogan wurde die Langzeitinternierung in der Klinik, die mit einem Verlust an Selbstbestimmung einhergeht, dem selbstbestimmten Leben in Wohngemeinschaften gegenübergestellt. Das Klinikbett oder dessen Nichtvorhandensein wird dabei zum Symbol für das (Über‑)Leben im Klinikalltag und für die damit verbundenen Einschränkungen von Selbstständigkeit und Selbstbestimmung. Demgegenüber werden Wohnen und das Gestalten von Wohnraum als befreiendes und selbstermächtigendes Moment vorgeführt.

Bei Protesten wird ein Raum geschaffen, in dem die ersehnten Verhältnisse bereits für einen kurzen Moment aufscheinen – hier das auf der Straße errichtete selbstbestimmte Wohnen außerhalb der Klinik. Die bei diesem Straßenfest erschaffene Zukunft wird als Teil der Stadt imaginiert und geht mit einer Aufforderung an deren Bewohner*innen einher, die ein zweites Banner mit der Aufschrift „Hallo Nachbar – Rückkehr ist machbar. Bremer aus Kloster Blankenburg wollen wieder zurück in die Gemeinde“ verkündet.^22^ Offenbar konnten sich die Bewohner*innen des Stadtteils Walle auf dem Fest bereits in diese Zukunft einschreiben. Unter den Bildern ist eine Petition mit einer Liste von Unterschriften abgedruckt. Sie trägt die Überschrift: „Wir [sic!] die zukünftigen Waller Nachbarn [sic!] unterstützen die Rückkehr der Blankenburger“.^23^ Es folgen zwei Seiten mit Unterschriften.

Diese Form des performativen Widerspruchs, durch Straßenprotest politische Teilhabe einzufordern, obwohl dazu nur unzureichende Möglichkeiten bestehen (Vogler 2020: 78), zeigt sich auch bei den Mad Pride-Paraden. 2021, als die Pride-Parade aufgrund der Corona-Auflagen online stattfinden musste,^24^ hielt die Künstlerin Faulenza eine Rede und musizierte. Sie erzählte den Zuschauer*innen von einem Tagtraum, den sie nach einer queeren Pride-Parade hatte. Er handelt von einem verrückten Aufstand in der betreuten WG, in der sie lebt:

„Ich träumte von einem Streik der Bewohner*innen meiner therapeutischen WG: behinderte Revolution, verrückter Aufstand. Wir Klient*innen von stationären Einrichtungen würden uns mit den Arbeitskämpfen der Betreuer*innen solidarisieren, die uns durch das tägliche Zusammenleben vertraut sind: höherer Lohn, mehr Urlaubstage, mehr Kolleg*innen pro Einrichtung für einen besseren Betreuungsschlüssel.“

Faulenza trägt auch Vorstellungen über einen veränderten, selbstbestimmten Tagesablauf, freie Zimmerwahl und größere Unabhängigkeit im betreuten Wohnen vor und fährt fort:

„Wir Bewohner*innen würden Sozialamt und Jugendamt erzittern lassen, denn wir würden auf deren Kostenübernahmeverfahren, Hilfekonferenzen und Gutachten scheißen. Warum? Niemand weiß besser als wir, wie lange und welche Hilfe wir brauchen. Und so genehmigen wir unsere Anträge einfach selbst“ (SO36 2021).

Faulenzas Traum von der Revolution beschreibt Herausforderungen aktueller sozial- und gemeindepsychiatrischer Einrichtungen. Er benennt prekäre Arbeitsbedingungen von Sozialarbeiter*innen, schildert das Leben der Bewohner*innen und Klient*innen als von Unsicherheit und Zwang geprägt und kritisiert die bürokratische Ordnung der Heime, angefangen vom strikten Tagesablauf bis zur Praxis von Gutachten und Kostenübernahmeverfahren (vgl. Weigand 2015). Doch über die Kritik des Bestehenden hinaus entwirft Faulenza einen Ausweg. In ihrer Rede transformiert sich der Aufstand in eine Tanzdemo mit Potenzial zur Aufregung:

„Aus dem Streik wurde in meiner Fantasie plötzlich die verrückteste Tanzdemo aller Zeiten. Der Medikamentenschrank würde geplündert und verteilt werden und aus den Boxen würde Krawall und Remmidemmi schallen. […] Behinderter Aufstand in ganz Berlin. Rasend würde er sich ausbreiten und schließlich auch die Psychiatriestationen erfassen. Nun gut. So oder so ähnlich würde das laufen. Zumindest in meiner Fantasie“ (ebd.).

Mit spielerischen Visionen eines anderen, befreiten Alltags entwirft Faulenza ein Bild der Zukunft, das deutlich macht, dass es in den Mad Pride-Paraden über Praktiken des Empowerments hinaus auch um das Abschaffen der Institution Psychiatrie – auch in seiner sozialpsychiatrisch reformierten Form – und einen veränderten Umgang mit psychischer Diversität geht (Maskos 2013). Eine andere Welt wird vorstellbar. Dabei wird zugleich das Gefühl der Gemeinsamkeit im Hier und Jetzt gestärkt und ein Handlungsziel – sei es auch noch so unerreichbar in weiter Ferne – formuliert, das die weitere politische Arbeit anleiten soll.

Obwohl beide Bewegungen auf eine mögliche Zukunft verweisen, wird durch das gemeinsame Erzählen der Visionen von Karawane und Mad Pride-Paraden auch ihr unterschiedlicher gesellschaftlicher Einfluss deutlich. Für die Blaue Karawane eröffnete sich tatsächlich ein Möglichkeitsraum, um die imaginierten Zukünfte in die Gegenwart vordringen zu lassen. Im Zuge der Auflösung von Blankenburg entstanden Wohngemeinschaften im Bremer Stadtteil Walle. Noch heute befindet sich in Walle das „Blaumeier Atelier“, das aus den Karawanen hervorging und in dem Menschen mit und ohne Behinderung und Psychiatrieerfahrung Theaterprojekte realisieren. Das integrative Wohnprojekt „Blauhaus“ mit der benachbarten „Blauen Manege“, die Werkstätten und Nachbarschaftstreff verbindet, ist Teil des noch heute bestehenden Vereins Blaue Karawane e. V. Dabei wird deutlich, dass die Blaue Karawane in der Bremer Stadtgesellschaft verankert ist, diese durch ihre Aktionen geprägt hat und weiterhin prägt. Auch der historische Moment der Entstehung der Karawane muss erneut betont werden: Im Zuge der Umsetzung der Psychiatriereformen in Bremen in den 1980er Jahren fand die Blaue Karawane einen Resonanzraum, in dem Veränderungen tatsächlich umgesetzt werden konnten, wenn auch nicht in der Radikalität, die sich die Akteur*innen der Blauen Karawane gewünscht hatten.

Heute sind die Forderungen nach einer Umstrukturierung der Psychiatrie in den Hintergrund gesellschaftlicher Debatten gerückt. Daher bleiben die Visionen der Mad Pride-Paraden oftmals in einem imaginativen Raum. Gegenwärtig haben sie nur wenig Handlungsmacht und treffen kaum auf Räume politischer Teilhabe. So erreicht der Protest der Mad Pride eher einen kleinen, vornehmlich gleichgesinnten Adressat*innenkreis. Dennoch stellen die Paraden Momente der Sichtbarkeit von Menschen mit Behinderungen, psychiatrischen Diagnosen und Erfahrungen mit psychiatrischer Gewalt dar und eröffnen Räume für Vernetzung. Sie insistieren darauf, in der Öffentlichkeit gesehen zu werden und beharren darauf, dass gesellschaftliche Verhältnisse verändert werden können.

## Von der Arbeit an der Multiplizität: Einige abschließende Überlegungen

Mit der Blauen Karawane und den Mad Pride-Paraden haben wir zwei Bewegungen zueinander in Relation gesetzt, die in Form öffentlicher Inszenierungen Kritik an der Psychiatrie und am gesellschaftlichen Umgang mit psychischer Alterität in den öffentlichen Raum tragen. Mit ihren Paraden und Reisen sensibilisieren sie ihr Publikum für die Gewaltförmigkeit psychiatrischer Praxis und deren Kategorisierungssysteme. Die jeweiligen Kritiken sind mit ihren spielerischen bis provokativen Interventionen und Interaktionsangeboten Teil dessen, was wir eingangs als Arbeit an der Multiplizität (Bodden 2022; Massey 2015) eingeführt haben: Die Forderung, psychische wie körperliche Alterität als selbstverständlichen Teil öffentlichen Lebens anzuerkennen, setzt an der etablierten Binarität normal#verrückt an. Dieser Fokus rückt andere Kategorisierungssysteme, die – wie etwa *class, race* und *gender* – machtvoll den öffentlichen Raum durchkreuzen, weitgehend in den Hintergrund. Der lange Zeitraum, den beide Bewegungen umfassen, zeigt einerseits die Persistenz, mit der etablierte Ordnungen des vermeintlich Normalen trotz Tendenzen der Flexibilierung sich behaupten. Andererseits weisen die Unterschiede zugleich auf Verschiebungen hin. Wir sind den jeweiligen Logiken in drei Schritten mit unterschiedlichen Schwerpunkten gefolgt.

Bei beiden Aktionsformen stehen die Besetzung des städtischen Raums und die öffentliche Artikulation von Kritik am gesellschaftlichen Umgang mit psychischer Alterität im Zentrum. Beide nutzen Formen des Spektakels, der Provokation und des Karnevalesken und zielen darauf ab, das Publikum mit zum Teil drastischen Mitteln aus Alltagsroutinen herauszureißen und für das zu sensibilisieren, was sonst meist im Verborgenen des klinischen oder sozialpsychiatrischen Alltags passiert oder im Getriebe „normalen“ großstädtischen Lebens untergeht. In den Aktionen sind jeweils auch transgressive Potenziale angelegt, die über die Gegenwart hinaus in eine bessere Zukunft weisen. Die Nähe der Mad Pride-Paraden zu den Mad Studies einerseits und die Verbindung institutioneller Psychiatriekritik mit gesellschaftlichen Diskussionen nach 1968 andererseits sorgen auch dafür, dass in den performativen Akten unterschiedliche Formen der Artikulation, unterschiedliche Bündnispolitiken und Potenzialitäten aufscheinen. Beiden Zeiträumen gemein ist jedoch die Politisierung der Unterscheidung normal#verrückt. Die Blaue Karawane schließt an Versuche an, den Umgang mit psychischer Alterität zu einer gesamtgesellschaftlichen Aufgabe zu erklären und somit alle – nicht zuletzt die Verantwortlichen in der Politik – in die Verantwortung zu nehmen. Während hier eine gesellschaftliche Gemeinschaft selbstverständlich adressiert wird, ist dies bei den Mad-Pride-Paraden komplizierter geworden. Das liegt auch an der grundsätzlichen Kritik von Kategorisierungen, wie sie in den Queer oder Mad Studies angelegt ist. Die Auseinandersetzung um ein gemeinschaftliches Wir ist von stetigen Diskussionen darüber bestimmt, Gemeinschaft in Differenz beziehungsweise über Differenzen hinweg herzustellen. Zwar bilden solidarische Momente das Zentrum der jeweils aufscheinenden Potenzialitäten der Aktionen. Gleichzeitig schränkt in beiden Zeiträumen die Fokussierung auf die Unterscheidung normal#verrückt die Aufmerksamkeit für weitere wirksame Differenzmarkierungen ein – auch wenn das Wissen um Differenz die Basis für gegenwärtige Debatten um Ein- und Ausschlüsse, aber auch für die Suche nach Möglichkeiten eines Gemeinsamen in der Differenz ist. Umso wichtiger ist es, dass die Visualität der Paraden Vielfalt sowie die Multiplizität von Positionen sichtbar und erfahrbar macht. Letztlich trägt das Schreiben über diese Paraden selbst dazu bei, diese Erfahrung verfügbar zu halten – als Wissen über soziale Kämpfe um Anerkennung, als Sensibilisierung für den Umgang mit psychischer Alterität und als Grundlage für die Weiterverhandlung von Multiplizität im städtischen Raum.

## Footnotes

1 Mit dem Asterisk (*) wird die Vielfalt an geschlechtlichen Identitäten zwischen und jenseits der Binarität von männlich und weiblich kenntlich gemacht.

2 Die Bezeichnung der Veranstaltungen variiert zwischen Mad Pride-Parade, Mad & Disability Pride Parade und Pride Parade. Wir werden sie generell Mad Pride-Paraden nennen, es sei denn, der Kontext verlangt eine andere Bezeichnung. Da der Fokus auf dem Umgang mit psychischer Alterität liegt, gehen wir auf Allianzen mit Behindertenbewegungen nur punktuell ein.

3 Kritik an der Psychiatrie gibt es nicht erst seit dem Ende des 20. Jahrhunderts. Vielmehr ist das Konzept „Irrenhaus“ seit dessen Entstehung von kritischen Stimmen begleitet, die konstitutiv für dessen Diskurs sind (vgl. z. B. Brückner 2022: 6).

4 Der Beitrag ist im Rahmen des DFG-Projekts „‚Antipsychiatrie‘ und Stadt. Zur Verschränkung von psychiatriekritischer Praxis, sozialen Bewegungen und städtischen Räumen“ (BI 1353/12‑1, im Rahmen der DFG-Forschungsgruppe Normal#verrückt: Zeitgeschichte einer erodierenden Differenz (Leitung: Prof. Dr. H. Fangerau, Universität Düsseldorf, Laufzeit 1. Projektphase 2021–2024) entstanden (siehe: https://www.normalverrueckt.hhu.de/). Die Ausführungen zur Blauen Karawane stützen sich auf Materialien des Berliner Archivs für Sozialpsychiatrie (BAS) und auf Dokumente in der Sammlung von Klaus Pramann (SKP), einem Aktivisten der Blauen Karawane, der seit den 1980er Jahren eine Vielzahl an Zeitungsartikeln, Sitzungsprotokollen und Veröffentlichungen der Gruppe privat gesammelt hat. Außerdem fließen Interviews mit Akteur*innen der Blauen Karawane in die Analyse ein. Die Untersuchungen der Mad Pride-Paraden gründen auf Literatur, Online-Präsentationen (z. B. https://www.pride-parade.de/, https://rausgegangen.de/events/mad-pride-0/) sowie auf Interviews mit Aktivist*innen der Mad Pride aus Deutschland und der Schweiz.

5 Die Schreibweise normal#verrückt soll auf die Brüchigkeit der Trennung zwischen normal und verrückt und auf das wechselseitige Angewiesensein der beiden Kategorien verweisen.

6 *Die Blaue Karawane kommt mit Marco Cavallo aus Triest, den Bremer Stadtmusikanten und vielen anderen*. Broschüre 1984, SKP.

7 *Die Blaue Karawane kommt *(siehe Anmerkung 8).

8 Zeitungsartikelsammlung, SKP.

9 Aufruf zum Sternmarsch 1980 von der Deutschen Gesellschaft für Soziale Psychiatrie (DGSP) 1980, Flyer. *Sternmarsch ’80 I*, BAS.

10 Basaglia (1973) arbeitete bereits als Direktor der psychiatrischen Klinik in Gorizia an einer Demokratisierung und Enthierarchisierung der Klinik.

11 Ein lebendiges Pferd namens Marco wurde in der Triester Anstalt zum Transport von Wäsche genutzt. Als Tier, das die Grenzen der Anstalt überschreiten konnte, wurde es zu einer Ikone. Als es alt und arbeitsuntüchtig geworden war, rettete eine Petition der Pfleger*innen, Ärzte und Patient*innen dem Pferd das Leben. Wiedererweckt wurde es im Rahmen eines Kunstprojekts als blaues Pferd, bestehend aus einem Holzrahmen, Pappmaché und Rollen (Voigtländer 2023: 5).

12 Die Kliniken in München-Haar, Wiesloch, Heidelberg, Herborn, Gütersloh, Bethel, Hamburg-Ochsenzoll, Blankenburg und Bremen-Ost wurden besucht.

13 Das Réseau-Alternative à la psychiatrie (Netzwerk Alternative für die Psychiatrie) wurde 1975 in Brüssel im Umfeld von Mony Elkaïm gegründet. Dabei waren unter anderem Giovanni Jervis, Félix Guattari, Robert Castel und Franco Basaglia beteiligt. Es richtete einige Jahre lang quasi weltweit Kongresse mit minoritären Initiativen aus, darunter von Gefangenen, Psychiatrieerfahrenen oder auch der Frauenbewegung (Elkaïm 1977).

14 Die zweite Blaue Karawane zum Thema Grenzüberschreitungen setzte sich 1994 von Leipzig bis Bremen für Ost-West-Begegnungen ein. Die dritte Blaue Karawane zog auf dem Wasser über Oldenburg nach Bremen und besuchte unterschiedliche Orte gesellschaftlicher Ausgrenzung (Gefängnis, ehemalige Psychiatrie, Geflüchtetenunterkunft). Die vierte Blaue Karawane trat als Schifffahrt unter dem Motto „Zum Glück geht es anders“ für die Revision gesellschaftlicher Normen und Regelungen ein (Blaue Karawane 2008: 1).

15 Auch wenn die Bezeichnung Mad Studies – wie auch die aktivistische Aneignung von *mad* als Label einer (stolzen) Selbstbezeichnung – umstritten ist (vgl. Beresford 2020), haben wir uns entschieden, den Begriff im Sinne der Veranstalter*innen zu übernehmen.

16 Noch expliziter geht das Projekt „Queere Bildungs- und Antidiskriminierungsarbeit in MV“ (Qube), das die Mad & Disability Pride in Greifswald organisiert, auf die vergeschlechtlichte Dimension psychiatrischer Diagnostik ein: „Als verrückt angesehen werden, hat für queere Menschen eine lange Tradition“ (Anonym 2022: 29).

17 Siehe die Redebeiträge unter *Behindert und verrückt feiern – Pride Parade Berlin* (2013b).

18 *Die Blaue Karawane kommt *(siehe Anmerkung 8).

19 Reise- und Programmplan für die Blaue Karawane, Flyer, 1985, SKP.

20 Ausstellung Blauhaus, Bremen: *Klosterbote, *2. Ausgabe, Dezember 1983. S. 8, SKP.

21 Ausstellung Blauhaus (siehe Anmerkung 20).

22 Ausstellung Blauhaus (siehe Anmerkung 20).

23 Ausstellung Blauhaus (siehe Anmerkung 20).

24 Uns ist bewusst, dass die Verbindung von Online- und Offline-Aktivismus differenzierter betrachtet werden müsste, wir können dies aber an dieser Stelle nicht leisten. Wir verweisen auf Hine (2015), die das dichte Verwobensein beider Räume und Praktiken hervorhebt.

## References

[CR1] Abrams, Benjamin und Peter Gardner 2023. Introducing Symbolic Objects in Contentious Politics. In: Benjamin Abrams und Peter Gardner (Hg.). *Symbolic Objects in Contentious Politics.* Ann Arbor, MI: University of Michigan Press: 1–12.

[CR2] Aehnelt, Reinhard 1982. Drei Jahrzehnte für den Frieden. Wie es zum Ostermarsch kam – Erinnerungen und Gespräche. In: Reinhard Aehnelt und Winfried Schwamborn (Hg.). *Wege zum Frieden. Die Ostermärsche*. Dortmund: Weltkreis: 14–35.

[CR4] Anonym 2022. Redebeitrag von Qube. In: Qube – Queere Bildungs- und Antidiskriminierungsarbeit in MV/Jugend kann bewegen e. V. (Hg.). *Zwischen(t)räume. Texte vom Disability & Mad Pride Festival*. URL: https://bildung-qube.de/wp-content/uploads/2023/01/Druckversion_Zwischentraeume-5.pdf Broschüre (o. D.): 27–32.

[CR5] Bachtin, Michail 1995. *Rabelais und seine Welt. Volkskultur als Gegenkultur*. Frankfurt am Main: Suhrkamp.

[CR6] Basaglia, Franco (Hg.) 1973. *Die negierte Institution oder Die Gesellschaft der Ausgeschlossenen. Ein Experiment der psychiatrischen Klinik in Görz. *Frankfurt am Main: Suhrkamp.

[CR9] Behindert und verrückt feiern – Pride Parade 2020. Der Pride-Parade-Film 2020. URL: https://pride-parade.de/blog/der-pride-parade-film-2020 (o.D.).

[CR7] Behindert und verrückt feiern – Pride Parade Berlin 2013a. *Aufruf 2013.* URL: https://pride-parade.de/die-parade/parade-2013/aufruf-2013 (o. D.).

[CR8] Behindert und verrückt feiern – Pride Parade Berlin 2013b. *Redebeiträge**2013.* URL: https://pride-parade.de/die-parade/parade-2013/redebeitraege-2013 (o. D.).

[CR10] Behindert und verrückt feiern – Pride Parade Berlin o. J. *FAQ.* URL: https://pride-parade.de/faq (o.D.).

[CR11] Beresford, Peter 2020. „Mad“, Mad studies and advancing inclusive resistance. *Disability & Society* (35/8):1337–1342. 10.1080/09687599.2019.1692168.

[CR12] Beresford, Peter und Jasna Russo (Hg.) 2021. *The Routledge International Handbook of Mad Studies*. London: Routledge.

[CR13] Besuden, Eike 1994. *Man müsste ne Klatsche haben, wa?* Radio Bremen. Film, 44 min., gepostet von user Blaue Karawane auf YouTube. https://www.youtube.com/watch?v=FjERlKODgEs (03.04.2018).

[CR14] Beyer, Christoph 2022. Radikale Psychiatriekritik und die Transformation des Anstaltswesens in der Bundesrepublik. In: Rudloff Wilfried, Franz-Werner Kersting, Marc von Miquel und Malte Thießen (Hg.). *Ende der Anstalten? Großeinrichtungen, Debatten und Deinstitutionalisierung seit den 1970er Jahren*. Paderborn: Brill Schöningh: 155–173.

[CR15] Blaue Karawane 2008. *Die Blaue Karawane, Zeitung für verrückte und andere Normale* 1. https://blauekarawane.de/wp-content/uploads/2023/01/zeitung_die_blaue_karawane_02.2008.pdf (o. D.).

[CR16] Blaue Karawane o. J. *Geschichte*. URL: https://blauekarawane.de/geschichte/ (o. D.).

[CR17] Bloch, Ernst 2016 [1935]. *Erbschaft dieser Zeit*. Frankfurt am Main: Suhrkamp.

[CR18] Blumental, Anne 2009. *Das Narrenschiff*. Film, 52 min, gepostet von user Blaue Karawane auf YouTube. https://www.youtube.com/watch?v=_nYvF5hORIw&t=897s (03.04.2018).

[CR19] Bodden, Shawn 2022. A work-in-progress politics of space. *City* (26/2–3): 397–410.

[CR20] Bravmann, Scott 2003. Queere Fiktionen von Stonewall. In : Andreas Kraß (Hg.): *Queer Denken*. Frankfurt a.M.: Suhrkamp: 240–274.

[CR21] Brückner, Burkhart 2022. „Schlammströme, in denen man uns ersaufen möchte“. Zur Genese des Begriffs „Antipsychiatrie“ im frühen 20. Jahrhundert. Sozialpsychiatrische Informationen (52/2): 6–10.

[CR22] Bryant, Rebecca und Daniel M. Knight 2019. *Anthropology of the Future*. Cambridge: Cambridge University Press.

[CR23] Chakkalakal, Silvy 2021. Figuration als Poiesis. Macht, Differenz und Ungleichheit in der figurationalen Kulturanalyse. In: Peter Hinrichs, Martina Röthl und Manfred Seifert (Hg.). *Theoretische Reflexionen: Perspektiven der Europäischen Ethnologie*. Berlin: Reimer: 135–151.

[CR24] Cremona, Vicki Ann 2018. *Carnival and Power. Play and Politics in Crown Colony*. Cham: Palgrave Macmillan.

[CR25] Denk, Larissa und Fabian Waibel 2009. Vom Krawall zum Karneval. Zur Geschichte der Straßendemonstration und der Aneignung des öffentlichen Raumes. In: Klaus Schönberger und Ove Sutter (Hg.). *Kommt herunter, reiht euch ein … Eine kleine Geschichte der Protestformen sozialer Bewegungen*. Berlin: Assoziation A: 46–81.

[CR26] Dörner, Klaus und Ursula Plog 1978. *Irren ist menschlich oder Lehrbuch der Psychiatrie/Psychotherapie. *Rehburg-Loccum: Psychiatrie-Verlag.

[CR27] Drebes, Sven und Friederike Jonah Reher 2021. Inklusiv gegen Exklusion demonstrieren. Erfahrungen aus der Organisation der „behindert und verrückt feiern“ Pride Parade. *Zeitschrift für Disability Studies *Nr. 1: 10.15203/ZDS_2021_1.06.

[CR28] Elkaïm, Mony (Hg.) 1977. *Réseau alternative à la psychiatrie: collectif international*. Paris: Union générale d’éditions.

[CR29] Fuchs, Petra 2022. „Behinderung“ – eine bewegte Geschichte. In: Anne Waldschmidt (Hg.). *Handbuch Disability Studies*. Wiesbaden: Springer Fachmedien: 35–53.

[CR30] Grosz, Elizabeth 2011. The Future of Feminist Theory: Dreams for New Knowledges. In: *Becoming Undone: Darwinian Reflections on Life, Politics, and Art*. New York: Duke University Press. 10.1215/9780822394433-006.

[CR31] Hine, Christine 2015. *Ethnography for the Internet: Embedded, Embodied and Everyday*. London: Bloomsbury.

[CR32] Hölling, Iris 2000. *Die Institution Psychiatrie in der Perspektive von Foucaults Machtkonzeptionen und die Relevanz von „Geschlecht“*. Berlin: Peter Lehmann Antipsychiatrieverlag.

[CR33] Ingram, Richard A. 2016. Doing mad studies. Making (non)sense together. *Intersectionalities. A Global Journal of Social Work Analysis, Research, Polity, and Practice *(5/3): 11–17.

[CR35] Jäger, Monika 1994. Im Banne der blauen Narren, *Mindener Tageblatt* 25.08.1994.

[CR34] Jäger, Margarete und Siegfried Jäger 2007. *Deutungskämpfe. Theorie und Praxis Kritischer Diskursanalyse*. Wiesbaden: VS Verlag für Sozialwissenschaften.

[CR36] Kraus, Dorothea 2007. Straßentheater als politische Protestform. In: Martin Klimke und Joachim Scharloth (Hg.). *1968 – Handbuch zur Kultur- und Mediengeschichte der Studentenbewegung*. Stuttgart: Springer: 89–100.

[CR37] Kruckenberg, Peter 1991. Mit Geld die Verbundenheit fördern. Zusammenhänge von politischen Reformentscheidungen, Finanzierung und Strukturreform. In: Petra Gromann (Hg.). *Was heißt hier Auflösung? Die Schließung der Klinik Blankenburg.* Bonn: Psychiatrie-Verlag.

[CR38] Küpper, Christian 2022. Immer diese Widersprüche. *Sozialpsychiatrische Informationen* (52/2): 34–36.

[CR39] Lang, Barbara 1998. *Mythos Kreuzberg. Ethnographie eines Stadtteils (1961–1995)*. Frankfurt am Main: Campus.

[CR40] Laufenberg, Mike 2019. Queer Theory: Identitäts- und machtkritische Perspektiven auf Sexualität und Geschlecht. In: Beate Kortendiek, Birgit Riegraf und Katja Sabisch (Hg.). *Handbuch Interdisziplinäre Geschlechterforschung*. Wiesbaden: Springer: 331–338.

[CR42] LeFrançois, Brenda A., Robert Menzies und Geoffrey Reaume (Hg.) 2012. *Mad Matters: A Critical Reader in Canadian Mad Studies*. Toronto: Canadian Scholars.

[CR41] LeFrançois, Brenda A., Peter Beresford und Jasna Russo 2016. Destination mad studies. *Intersectionalities. A Global Journal of Social Work Analysis, Research, Polity, and Practice *(5/3): 1–10.

[CR43] Lüthi, Eliah 2019. PsychGewalt_ig. In: Esto Mader, Cora Schmechel, Alex Steinweg und Kim Kawalska (Hg.). *Gegendiagnose II*. Münster: edition assemblage: 214–233.

[CR44] Lüthi, Eliah 2022. Mad Studies und Disability Studies. In: Anne Waldschmidt (Hg.). *Handbuch Disability Studies*. Wiesbaden: Springer: 435–452.

[CR45] Maskos, Rebecca 2013. Lass zucken! In: Jungle World Nr. 28.

[CR46] Massey, Doreen 2015 [2005]. *For Space*. London: Sage.

[CR47] McRuer, Robert 2023. Zwangsabilität und queere/behinderte Existenz. In: Mike Laufenberg und Ben Trott (Hg.). *Queer Studies. Schlüsseltexte*. Berlin: Suhrkamp: 247–263.

[CR48] Möller, Ludger 1991. Die Erfahrungen der Enthospitalisierungsstation. In: Petra Gromann-Richter (Hg.). *Was heißt hier Auflösung? Die Schließung der Klinik Blankenburg*. Bonn: Psychiatrie-Verlag: 39–45.

[CR49] Muñoz, José Esteban 2009. *Cruising Utopia. The then and there of Queer futurity*. New York: New York University Press.

[CR51] Pfahl, Lisa und Swantje Köbsell 2016. Von „Krüppelfrauengruppen“ zur „Disability and Mad Pride“: Grenzen der Sichtbarkeit von Geschlecht und Behinderung. In: Bauschke-Urban, Carola, Göde Both, Sabine Grenz, Inka Greusing, Tomke König, Lisa Pfahl, Katja Sabisch, Monika Schröttle und Susanne Völker (Hg.). *Bewegung/en: Beiträge zur 5. Jahrestagung der Fachgesellschaft Geschlechterstudien.* Leverkusen: Budrich: 61–74.

[CR50] Pramann, Klaus 1990. Mehr Psychiatrie statt weniger. *Alternative kommunale Politik, Fachzeitschrift für grüne und alternative Kommunalpolitik *Nr. 3: 40–43.

[CR3] AK Psychiatriekritik 2015. Der AK Psychiatriekritik – wider die psychiatrische Macht. In: Cora Schmechel, Fabian Dion, Kevin Dudek und Mäks* RoBmöller (Hg.). *Gegendiagnose*. Münster: edition assemblage: 289–312.

[CR52] Reumschüssel-Wienert, Christian 2021. *Psychiatriereform in der Bundesrepublik Deutschland. Eine Chronik der Sozialpsychiatrie und ihres Verbandes – der DGSP*. Bielefeld: transcript.

[CR53] Röhm, Elisabeth 1985. Große Gemeinschaft unterwegs? In: Martin Scharfe, Martin Schmolze und Gertrud Schubert (Hg.). *Wallfahrt. Tradition und Moderne.* Tübingen: Tübinger Vereinigung für Volkskunde: 83–88.

[CR54] Rudloff, Wilfried, Franz-Werner Kersting, Marc von Miquel und Malte Thießen 2022. Krise der Anstalten, Deinstitutionalisierung und gesellschaftlicher Wandel in Deutschland. In: dies. (Hg.). *Ende der Anstalten? Großeinrichtungen, Debatten und Deinstitutionalisierung seit den 1970er Jahren*. Paderborn: Brill Schöningh: 2–34.

[CR55] Russo, Jasna 2012. Survivor-Controlled Research: A New Foundation for Thinking About Psychiatry and Mental Health. *Forum: Qualitative Research *(13/1), Art. 8: 10.17169/fqs-13.1.1790.

[CR56] Russo, Jasna und Iris Hölling 2022. Leerstellen der gegenwärtigen Psychiatriekritik. *Sozialpsychiatrische Informationen (*52/2): 30–33.

[CR57] Schmuhl, Hans-Walter 2022. Der Anfang vom Ende der Anstalt. Impulse zur Psychiatriereform in den v. Bodelschwinghschen Anstalten Bethel in den frühen 1970er Jahren. In: Rudloff Franz-Werner Kersting, Marc von Miquel und Malte Thießen (Hg.). *Ende der Anstalten? Großeinrichtungen, Debatten und Deinstitutionalisierung seit den 1970er Jahren*. Paderborn: Brill Schöningh: 223–248.

[CR58] Schönberger, Klaus und Ove Sutter (Hg.) 2009. *Kommt herunter, reiht euch ein … Eine kleine Geschichte der Protestformen sozialer Bewegungen*. Berlin: Assoziation A.

[CR59] Schuster, Nina 2016. Ethnografische Zugänge zu einem queeren Raumverständnis. In: Barbara Paul und Lüder Tietz (Hg.). *Queer as … – Kritische Heteronormativitätsforschung aus interdisziplinärer Perspektive*. Bielefeld: transcript: 147–168.

[CR60] SO36 2021. Parade-Stream – Behindert und verrückt durch die Pandemie – Online statt Straße. Stream von user SO36 auf YouTube. https://www.youtube.com/watch?v=edL97cqGsKE (10.10.2021).

[CR61] Sommerblut 2021. Mad Pride-Parade Köln. Kompetenzzentrum Selbstbestimmt Lernen Regierungsbezirk Köln. URL: https://ksl-koeln.de/de/node/3852 (24.05.2021).

[CR62] Spandler, Helen und Meg-John Barker 2016. Mad and Queer studies: interconnections and tensions. *Mad Studies Network. *URL: https://madstudies2014.wordpress.com/2016/07/01/mad-and-queer-studies-interconnections-and-tensions/ (01.07.2016).

[CR63] Spivak, Gayatri Chakravorty 1988. Can the Subaltern Speak? In: Cary Nelson und Lawrence Grossberg (Hg.). *Marxism and the Interpretation of Culture*. Urbana, IL: University of Illinois Press, 271–313.

[CR64] Superbass 2016. *Bed Push mit dargestellter Fixierung und Zwangsbehandlung während der Mad Pride-Parade in Köln.* Fotografie von user Superbass in der Wikipedia. URL: https://de.wikipedia.org/wiki/Mad_Pride#/media/Datei:2016-05-16-Mad_Pride_K%C3%B6ln_2016-5001.jpg (16.06.2016).

[CR65] Tang, Lynn 2021. Upcycling Recovery. Potential alliances of recovery, inequality and Mad Studies. In: Peter Beresford und Jasna Russo (Hg.). *The Routledge International Handbook of Mad Studies*. London: Routledge: 266–275.

[CR67] Theatre du pain o. J. *Geschichte*. URL: http://www.theatredupain.de/2010/sites/geschichte.html (o. D.).

[CR66] Tietz, Lüder 2016. Pride-Paraden von LSBT*I*/Q. Möglichkeiten und Grenzen der Politik des Performativen. In: Barbara Paul und Lüder Tietz (Hg.). *Queer as … – Kritische Heteronormativitätsforschug aus interdisziplinärer Perspektive*. Bielefeld: transcript: 193–224.

[CR68] Toronto Mad Pride 2016. *Madness on Parade – Mad Bed Push. *URL: http://www.torontomadpride.com/2016/03/mad-pride-parade-bed-push/ (04.10.2024).

[CR69] von Trotha, Thilo 1993. Persönliche Beweggründe für antipsychiatrisches Handeln. In: Kerstin Kempker und Peter Lehmann (Hg.). *Statt Psychiatrie*. Berlin: Peter Lehmann Antipsychiatrieverlag: 413–417.

[CR70] von Trotha, Thilo 2001. Unterwegs zu alten Fragen. Die neue Antipsychiatrie. In: Karin Roth (Hg.). *Antipsychiatrie. Sinnerzeugung durch Entfesselung der Vielstimmigkeit*. Dortmund: verlag modernes lernen: 201–210.

[CR71] Van Veen, Christopher, Mohamed Ibrahim und Marina Morrow 2018. Dangerous Discourses: Masculinity, Coercion, and Psychiatry. In: Jennifer Kilty und Erin Dej (Hg.). *Containing madness: gender and *“*psy” in institutional contexts*. Cham: Palgrave Macmillan: 241–265.

[CR72] Vogler, Tanja 2020. Pride-Paraden. Queere Erinnerungspolitiken. In: Verena Sperk, Sandra Altenberger, Katharina Lux und Tanja Vogler (Hg.). *Geschlecht und Geschlechterverhältnisse bewegen. Queer/Feminismen zwischen Widerstand, Subversion und Solidarität*. Bielefeld: transcript: 73–95.

[CR73] Voigtländer, Wolfram 2023. Zur Titelabbildung. Freiheit heilt. Oder: Die einzige Alternative zum Irrenhaus ist seine Abschaffung. *Sozialpsychiatrische Informationen* (53/2): 4–6.

[CR74] Waldschmidt, Anne 1998. Flexible Normalisierung oder stabile Ausgrenzung: Veränderungen im Verhältnis Behinderung und Normalität. *Soziale Probleme* 9 (1): 3–25.

[CR75] Weigand, Stephan 2015. lnklusiv und repressiv. Zur Herrschaftsförmigkeit der reformierten Psychiatrie. In: Cora Schmechel, Fabian Dion, Kevin Dudek und Mäks* RoBmöller (Hg.). *Gegendiagnose*. Münster: edition assemblage: 20–46.

[CR76] Yates, Luke 2021. Prefigurative Politics and Social Movement Strategy: The Roles of Prefiguration in the Reproduction, Mobilisation and Coordination of Movements. *Political Studies* (69/4): 1033–1052.

